# Prognostic value of lymphocyte to monocyte ratio in pancreatic cancer: a systematic review and meta-analysis including 3338 patients

**DOI:** 10.1186/s12957-020-01962-0

**Published:** 2020-07-25

**Authors:** Shuwen Lin, Yinghua Fang, Zhikang Mo, Ye Lin, Chenggang Ji, Zhixiang Jian

**Affiliations:** 1 Department of General Surgery, Binhaiwan Central Hospital of Dongguan (also called The Fifth People’s Hospital of Dongguan), The Dongguan Affiliated Hospital of Medical College of Jinan University, Dongguan, 523905 Guangdong People’s Republic of China; 2 Department of Pain, Binhaiwan Central Hospital of Dongguan (also called The Fifth People’s Hospital of Dongguan), The Dongguan Affiliated Hospital of Medical College of Jinan University, Dongguan, 523905 Guangdong People’s Republic of China; 3 Department of General Surgery, Guangdong Provincial People’s Hospital, Guangdong Academy of Medical Sciences, No. 106 Zhongshan Er Road, Yuexiu, Guangzhou, 510080 Guangdong People’s Republic of China

**Keywords:** Lymphocyte to monocyte ratio, Pancreatic cancer, Prognosis, Meta-analysis

## Abstract

**Background:**

Recently, reports have classified lymphocyte to monocyte ratio (LMR) as an effective indicator for predicting the prognosis of pancreatic cancer. Nevertheless, the prognostic value of LMR for pancreatic cancer remains controversial. Through meta-analysis, this work intends to evaluate the potential prognostic role of pretreatment LMR in patients diagnosed with pancreatic cancer.

**Methods:**

We reviewed and extracted eligible articles from Web of Science, PubMed, Cochrane Library, and Embase. A meta-analysis was conducted using hazard ratio (HR) and 95% confidence intervals (CIs) to assess the comparison between pretreatment LMR and overall survival (OS) and disease-free survival/recurrence-free survival/time to progression (DFS/RFS/TTP).

**Results:**

In total, 11 studies (16 cohorts) including 3338 patients diagnosed with pancreatic cancer (PC) were enrolled in our meta-analysis. Notably, we revealed that high pretreatment LMR predicted better overall survival (OS) (HR = 0.68, 95% CI 0.58–0.80, *P* < 0.001, *I*-squared = 69.3%, Ph < 0.001) and DFS/RFS/TTP (HR = 0.55, 95% CI 0.31–0.96, *P* = 0.037, *I*-squared = 89.9%, Ph < 0.001) in patients with pancreatic cancer. Further, through subgroup analyses, we showed that high pretreatment LMR was significantly associated with the favorable OS regardless of ethnicity, study design, treatment method, variable type, the cut-off value for LMR, and disease stages of I–IV and III–IV.

**Conclusion:**

The findings from our study suggest that high pretreatment LMR is associated with better OS and DFS/RFS/TTP in patients diagnosed with pancreatic cancer. As such, it can potentially serve as a novel prognostic biomarker for patients with pancreatic cancer.

## Background

Pancreatic cancer (PC) is among the most aggressive malignant tumors and the 7th deadliest type of cancer globally with 5-year survival rate of less than 5% [1]. While acknowledging the increasingly advanced research on the diagnosis and treatment, the prognosis of PC remains unsatisfactory [2–4]. Moreover, despite surgical resection being the first choice in treating patients with PC, only 20% of these patients are diagnosed at an early stage [5, 6]. The continuous poor prognosis of PC patients may be attributed to the complex chemoresistance mechanisms and invasive phenotype as well as the important role of hypoxia in pancreatic cancers. Of note, clinically available tumor biomarkers are helpful in diagnosis, prognosis, and evaluation of treatment response in patients with digestive system’s tumors. There is a general increase in serum levels of CEA, CA19-9, CA12-5, pancreatic oncofetal antigen, and tissue polypeptide antigen (TPA) in PC patients [7]. In as much as these biomarkers are useful for monitoring tumor diseases, their levels may be elevated in patients with benign pancreatic diseases (BPD) [8, 9]. Being the most widely studied biomarker, CA19-9 is highly significant for diagnosis and assessing tumor stage, resectability, treatment response, and prognosis of patients with PC [10–13]. Nevertheless, there are some limitations to CA19-9 in clinical application, including limited sensitivity, increased false positives results during cholestasis, and false-negative results for Lewis negative phenotype [10]. Therefore, the high mortality rate in PC patients is partly attributed to the lack of sufficient prognostic biomarkers for predicting treatment response and survival. It is thus necessary to identify valuable serum biomarkers, which will help clinicians efficiently design individual treatment strategies for PC patients.

Currently, increasing evidence shows that systemic inflammation is closely linked to angiogenesis and cancer progression [14–17]. Additionally, inflammatory factors including lymphocyte to monocyte ratio (LMR), platelet to lymphocyte ratio (PLR), neutrophil to lymphocyte ratio (NLR), and C-reactive protein have been reported to be associated with prognosis of patients with various types of tumors [18–22]. In particular, the lymphocytes to monocytes ratio (LMR) has been associated with the overall survival of multiple malignant tumors among them, colon cancer, soft tissue sarcoma, and nasopharyngeal carcinoma [23–25]. Recently, several studies suggested that elevated pretreatment LMR can potentially predict better overall survival outcomes in patients with PC [26–28]. However, other research findings argue that the prognostic significance of pretreatment LMR in PC patients is still elusive [29–31]. Despite previous meta-analyses reporting the existing association between pretreatment LMR and survival outcomes in patients with PC [32, 33], more consideration by the preceding investigations is essential. Therefore, we aimed to conduct a systematic review and meta-analysis to comprehensively evaluate the prognostic value of pretreatment LMR in patients with PC using the latest data from current studies [29, 30, 34].

## Materials and methods

### Search strategy

This analysis was performed according to the PRISMA statement [35]. We retrieved comprehensive literature published until March 2019 from Web of Science, PubMed, Embase, and The Cochrane Library databases. The following free words were used as search subjects and titles: “Pancreas,” “pancreatic,” “neoplasms,” “carcinoma,” “cancer,” “malignancy,” “lymphocyte monocyte ratio,” “lymphocyte to monocyte ratio,” “LMR,” “lymphocyte-to-monocyte ratio,” “lymphocyte-monocyte ratio,” “prognosis,” “outcome,” and “survival”. Further, relevant references were retrieved conducting a manual search on eligible articles.

### Inclusion and exclusion criteria

Inclusion criteria included (1) PC patients were confirmed by pathological examination; (2) pretreatment LMR was detected from serum; (3) studies described the association of pretreatment LMR with disease-free survival (DFS), progression-free survival (PFS) or time to progression (TTP), and overall survival (OS); (4) HR with 95% CI was reported for data calculation; and (5) the cut-off value of LMR was described. Exclusion criteria were as follows: (1) case reports, letters, abstracts, review articles, editorials, and expert opinions; (2) data were republished in duplicates and repeatedly analyzed; (3) studies lacking sufficient information to assess HR and 95% CIs; and (4) non-English articles.

### Data extraction and quality assessment

All eligible studies were independently reviewed and extracted by two authors (Shuwen Lin and Yinghua Fang). Articles that could not be judged using the title and abstract were further evaluated by full-text. If contrary opinions emerged, the two authors would further discuss and reach a consensus with the inclusion of a third author (Zhikang Mo). For each eligible study, the information was gathered as follows: the first author, country, ethnicity, publication year, sample size, age, follow-up time, cut-off value, study design, treatment method, variable type, tumor stage, survival outcome, and HRs with 95% CIs. Taking into account the confounding factors of each study, HRs were extracted from multivariate analysis. However, if a multivariable analysis was not conducted, HRs were extracted from univariable analysis. The Newcastle-Ottawa Scale (NOS) was adopted to validate the methodological quality of each eligible study by two independent reviewers (Shuwen Lin and Yinghua Fang). The NOS includes the following components: selection of patient (0–4 points), comparability of groups (0–2 points), and outcome assessment (0–3 points). If the validation shows the NOS scores at ≥ 6, the studies would be regarded as high quality.

### Statistical analysis

The STATA statistical software version 15.1 (STATA, College Station, TX) was to analyze all statistical data. HR and 95% CI were directly extracted from each article or calculated following the methods described by Parmar et al. [36]. To perform the heterogeneity test, we used Cochran’s *Q*-statistics test and the *I*-squared statistic. To further process the data, a random-effects model was adopted. The pooled HRs and 95% CIs were applied to assess the prognostic value of LMR for OS and DFS/PFS/TTP. We further conducted subgroup analyses based on the following: cut-off value for LMR, ethnicity, study design, variable type, treatment, and tumor stage. Sensitivity analysis was used to estimate the stability of the results. Importantly, to establish any publication bias, we performed Begg’s funnel plot and Egger’s linear regression test. All statistical tests were two-tailed, and *P* < 0.05 was considered statistically significant.

## Results

### Search results

**Fig. 1:**
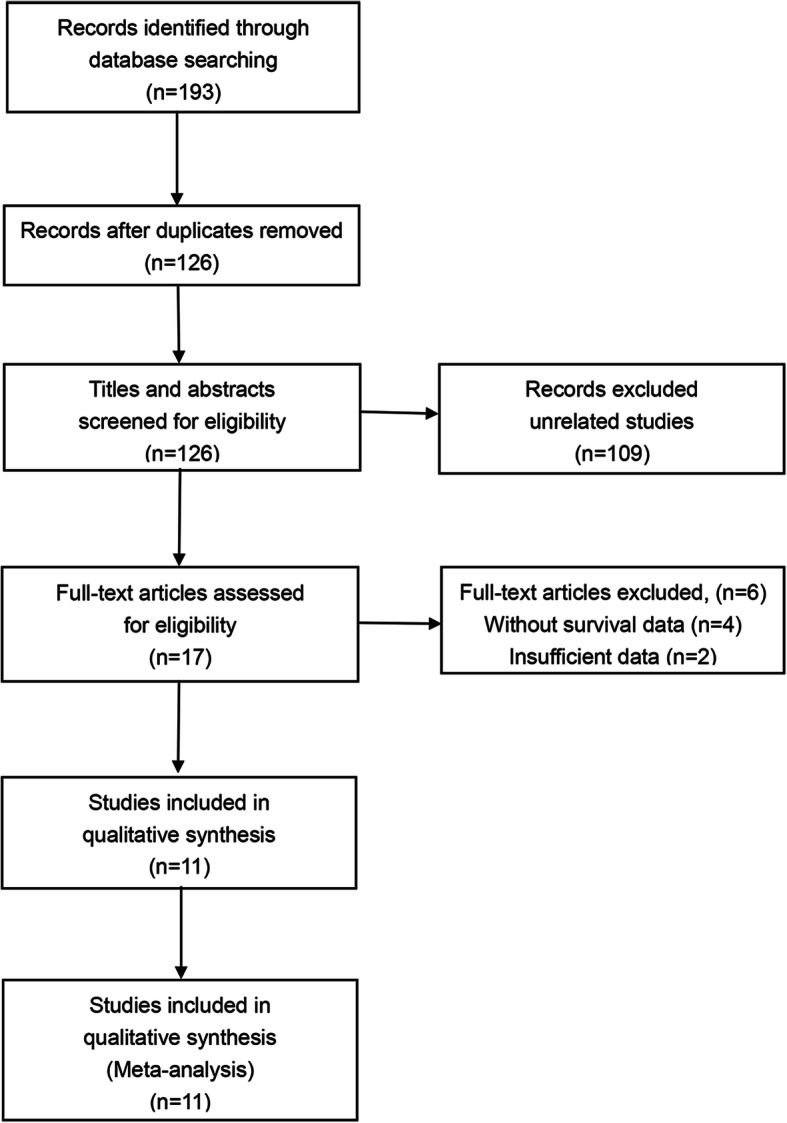
Flow chart of the included studies

Following the retrieval strategies stipulated above, we identified a total of 193 articles. Then, after a careful review of all the articles, 11 articles (16 cohorts) with 3338 patients published between 2015 and 2019 were enrolled for meta-analysis for further evaluation [26–31, 34, 37–40]. The selection process of articles is shown in Fig. 1 as per the Preferred Reporting Items for Systematic Reviews and Meta-Analyses (PRISMA) statement.

### Characteristics of the enrolled studies

In total, 3338 patients from 16 enrolled cohorts were included in this meta-analysis. Surprisingly, there were other two studies reported by (Qi et al.) from the same research center in China; however, their data were not replicated [31, 37]. According to the enrolled studies, patients from 13 cohorts were Asians, 2 Caucasians, and 1 were mixed Caucasians and Blacks. Again, 5 studies had been conducted in China, 4 from Japan, with 1 from the USA, Poland, and Austria, respectively. Toshiya’s, Xue’s, and Yu’s data were from 2 cohorts of different populations classified as into Toshiya (1), Toshiya (2), Xue (1), Xue (2), Yu (1), and Yu (2), respectively. Qi’s data in 2016 were from 3 cohorts of different populations and classified into Qi (1), Qi (2), and Qi (3). The HRs and 95% CIs of data presented in these studies were obtained through multivariable and/or univariable analysis. Among them, in 12 cohorts, HR was calculated through multivariable analysis whereas, in 4 cohorts, univariable analysis was used. The total number of enrolled patients ranged from 67 to 474. Cut-off values of LMR ranged from 2.05 to 4.6 where the LMR cut-off values of 11 cohorts were ≤ 3 whereas those of 5 cohorts were > 3. All the cohorts explored the OS while only 5 cohorts reported DFS/RFS/TTP. The NOS scores counted from 5 to 6. The parameters of all included cohorts are presented in Table 1.

**Table 1:** Main characteristics of all the studies included in the meta-analysis (no. = 3338)

Author	Year	Country	Ethnicity	No. (M/F)	Follow-up (months) (median and range)	Treatment	Age (years; mean ± SD)	Cut-off value	Outcome	Stage	Type	Variable type	NOS score
Manabu et al. [30]	2019	Japan	Asian	67 (40/27)	NR	Mix	68 (46–80)	4	OS	IIa–III	PC	UV	6
Shinichi et al. [34]	2019	Japan	Asian	136 (76/60)	16.8 (1.3–104.3)	Surgery	68 (33–86)	4.6	OS	I–IV	PDAC	UV	5
Toshiya et al. (1) [29]	2018	Japan	Asian	329 (131/198)	NR	Surgery	67 (61–74)	3	OS/DFS	0–III	PDAC	UV	5
Toshiya et al. (2) [29]	2018	Japan	Asian	95 (39/56)	NR	Surgery	65 (58–69)	3	OS	–	PDAC	UV/MV	5
Sierzega et al. [40]	2017	Poland	Caucasian	442 (182/260)	93 (15–290)	Surgery	60 (55–66)	3	OS	IA–III	PDAC	UV/MV	5
Singh et al. [27]	2017	USA	Mixed	97 (97/0)	NR	Mixed	66 ± 0.9	2.05	OS	I–IV	PDAC	UV	6
Xue et al. (1) [39]	2017	China	Asian	153 (102/51)	8.8 (0.5–75.5)	Chemotherapy	60 (34–86)	2.8	OS	III–IV	PDAC	UV/MV	6
Xue et al. (2) [39]	2017	Japan	Asian	252 (133/119)	NR	Chemotherapy	67 (31–86)	2.8	OS	III–IV	PDAC	UV/MV	6
Yu et al. (1) [38]	2017	China	Asian	139 (83/56)	78	Chemotherapy	< 60, 54 ≥ 60, 85	3.19	OS	III–IV	PDAC	UV/MV	5
Yu et al. (2) [38]	2017	China	Asian	225 (146/79)	78	Chemotherapy	< 60, 78 ≥ 60, 147	3.19	OS	III–IV	PDAC	UV/MV	5
Li et al. [26]	2016	China	Asian	144 (77/67)	14 (6–40)	Surgery	62 ± 2.8	2.86	OS/RFS	I–III	PDAC	UV/MV	6
Qi et al. (1) [31]	2016	China	Asian	177 (108/69)	NR	Chemotherapy	58.8 ± 10.7	3	OS/TTP	III–IV	PDAC	UV/MV	6
Qi et al. (2) [31]	2016	China	Asian	321 (208/113)	NR	Chemotherapy	61.0 ± 10.1	3	OS/TTP	III–IV	PDAC	UV/MV	6
Qi et al. (3) [31]	2016	China	Asian	76 (46/30)	NR	Chemotherapy	60.9 ± 9.6	3	OS/TTP	III–IV	PDAC	UV/MV	6
Qi et al. [37]	2015	China	Asian	211 (134/77)	NR	Chemotherapy	61.2 ± 10.7	3.33	OS	III–IV	PDAC	UV/MV	6
Stotz et al. [28]	2015	Austria	Caucasian	474 (256/218)	36 (0–162)	Mix	64.6 ± 10.4	2.8	OS	I–IV	PDAC	UV/MV	6

*OS* overall survival, *PFS* progression-free survival, *DFS* disease-free survival, *HR* hazard ratio, obtained by reporting in text (R), *MV* the HR come from multivariate analysis, *UV* the HR comes from univariate analysis; *Mix* mixed treatment including chemotherapy, surgery, and radiotherapy, *NR* not reported, *NOS* Newcastle–Ottawa Quality Assessment Scale, *PDA* pancreatic ductal adenocarcinoma

### LMR and OS in pancreatic cancer

**Fig. 2:**
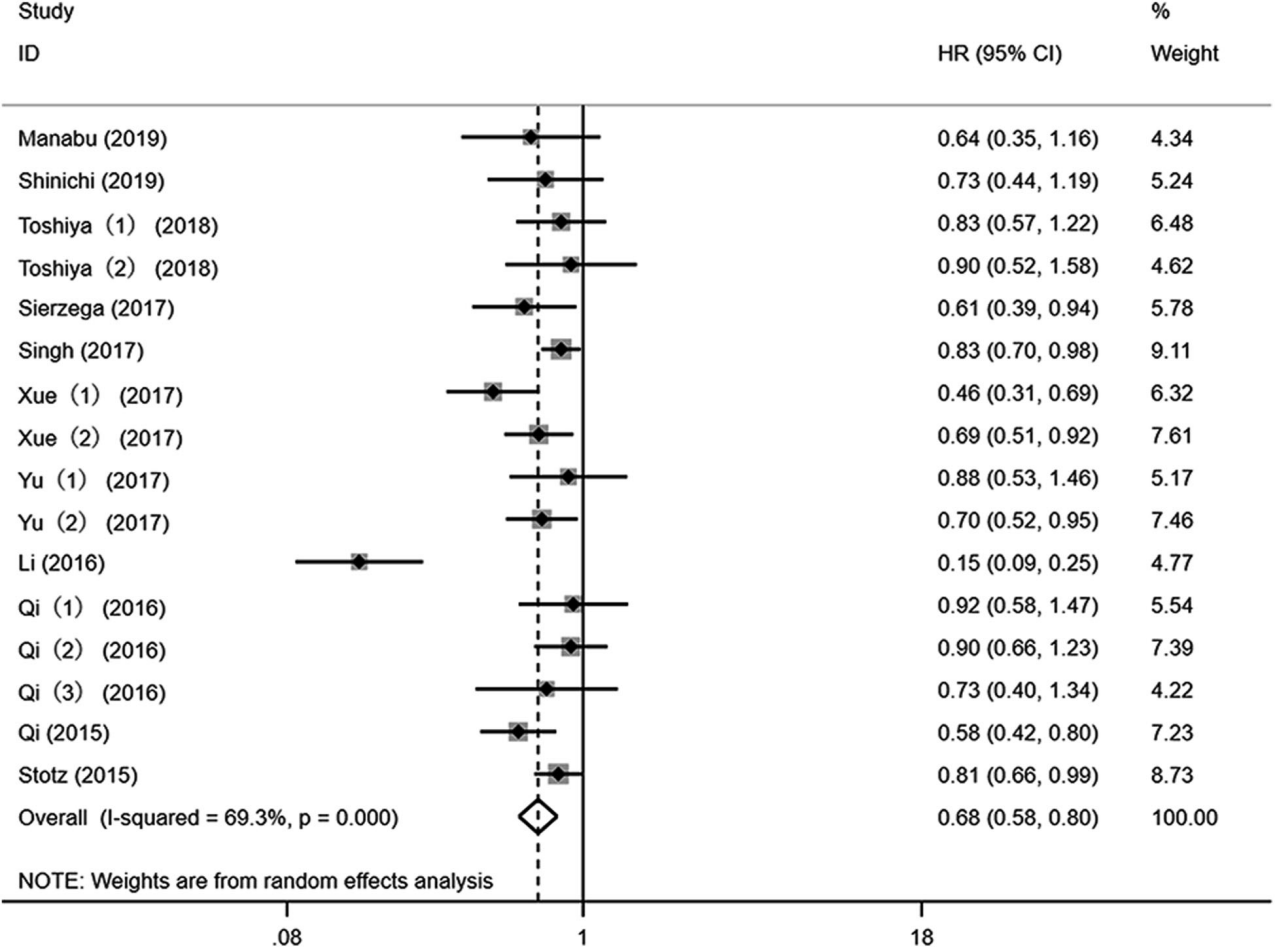
A meta-analysis of the association between pretreatment LMR and overall survival (OS) of pancreatic cancer. Results are presented as individual and pooled hazard ratios (HRs) and 95% confidence intervals (CIs)

In total, 11 studies (16 cohorts) assessed the association between LMR and OS in patients with PC. Results demonstrated that the heterogeneity was significant; therefore, a random-effects model was adopted (*I*-squared = 69.3%, Ph < 0.001). Data from pooled analyses revealed that elevated LMR predicted a better OS (HR = 0.68, 95% CI 0.58–0.80, *P* < 0.001) (Fig. 2).

To minimize the influence of literature heterogeneity, we conducted subgroup analyses based on the cut-off value of LMR, ethnicity, study design, treatment, variable type, and tumor stage (Table 2). The results demonstrated that LMR was significantly associated with OS in patients with PC regardless of ethnicity (Asian and Caucasian), study design (prospective and retrospective), treatment method (surgery, chemotherapy, and mixed), variable type (univariable and multivariable), and cut-off value (≤ 3 and > 3). However, for the tumor stage, the subgroup analyses revealed that LMR was a prognostic factor in patients with stage of III–IV (HR = 0.70, 95% CI 0.60–0.81, *P* < 0.001, random-effects model, *I*^2^ = 22.8%) and I–IV (HR = 0.82, 95% CI 0.72–0.92, *P* = 0.001, random-effects model, *I*^2^ = 0%).

**Table 2:** Subgroup analyses for the association between LMR and OS in PC

Subgroup	No of studies	No of patients	Effects model	HR (95% CI)	*P* value	Heterogeneity	
						*I*^2^ (%)	Ph
Overall	16	3338	Random	0.68 (0.58–0.80)	< 0.001	69.3	< 0.001
Ethnicity							
Asian	13	2325	Random	0.66 (0.53–0.82)	< 0.001	72.0	< 0.001
Caucasian	2	916	Random	0.75 (0.59–0.96)	0.025	26.3	0.244
Study design							
Prospective	8	1858	Random	0.73 (0.65–0.83)	< 0.001	25.5	0.226
Retrospective	8	1480	Random	0.64 (0.45–0.92)	0.015	81.7	< 0.001
Treatment							
Surgery	5	1146	Random	0.55 (0.30–0.99)	0.049	87.1	< 0.001
Chemotherapy	8	1554	Random	0.71 (0.61–0.83)	< 0.001	31.8	0.174
Mixed	3	638	Random	0.81 (0.72–0.92)	0.001	0	0.712
Variable type							
Univariate	4	629	Random	0.81 (0.70–0.93)	0.004	0	0.829
Multivariate	12	2709	Random	0.65 (0.52–0.81)	< 0.001	75.5	< 0.001
Cut-off for LMR							
≤ 3	11	2560	Random	0.67 (0.54–0.84)	< 0.001	78.2	< 0.001
> 3	5	778	Random	0.68 (0.57–0.81)	< 0.001	0	0.697
Tumor stage							
I–III	3	915	Random	0.43 (0.16–1.11)	0.081	92.5	< 0.001
III–IV	9	1621	Random	0.70 (0.60–0.81)	< 0.001	22.8	0.241
I–IV	3	707	Random	0.82 (0.72–0.92)	0.001	0	0.878

Both univariable and multivariable analyses were adopted in 10 cohorts. We then performed a meta-analysis according to the variable type of the same study, respectively. Based on the univariable analyses, the pooled results demonstrated that elevated pretreatment LMR was associated with better OS in patients with PC (HR = 0.57, 95% CI 0.50–0.65, *P* < 0.001, random-effects model, *I*-squared = 35.2%, Ph = 0.127) (Fig. S1). Whereas with multivariable analyses, the pooled results revealed that high pretreatment LMR implied longer OS in PC patients (HR = 0.75, 95% CI 0.66–0.84, *P* < 0.001, random-effects model, *I*-squared = 14.5%, Ph = 0.31) (Fig. S2).

### LMR and PFS/DFS/TTP in pancreatic cancer

**Fig. 3:**
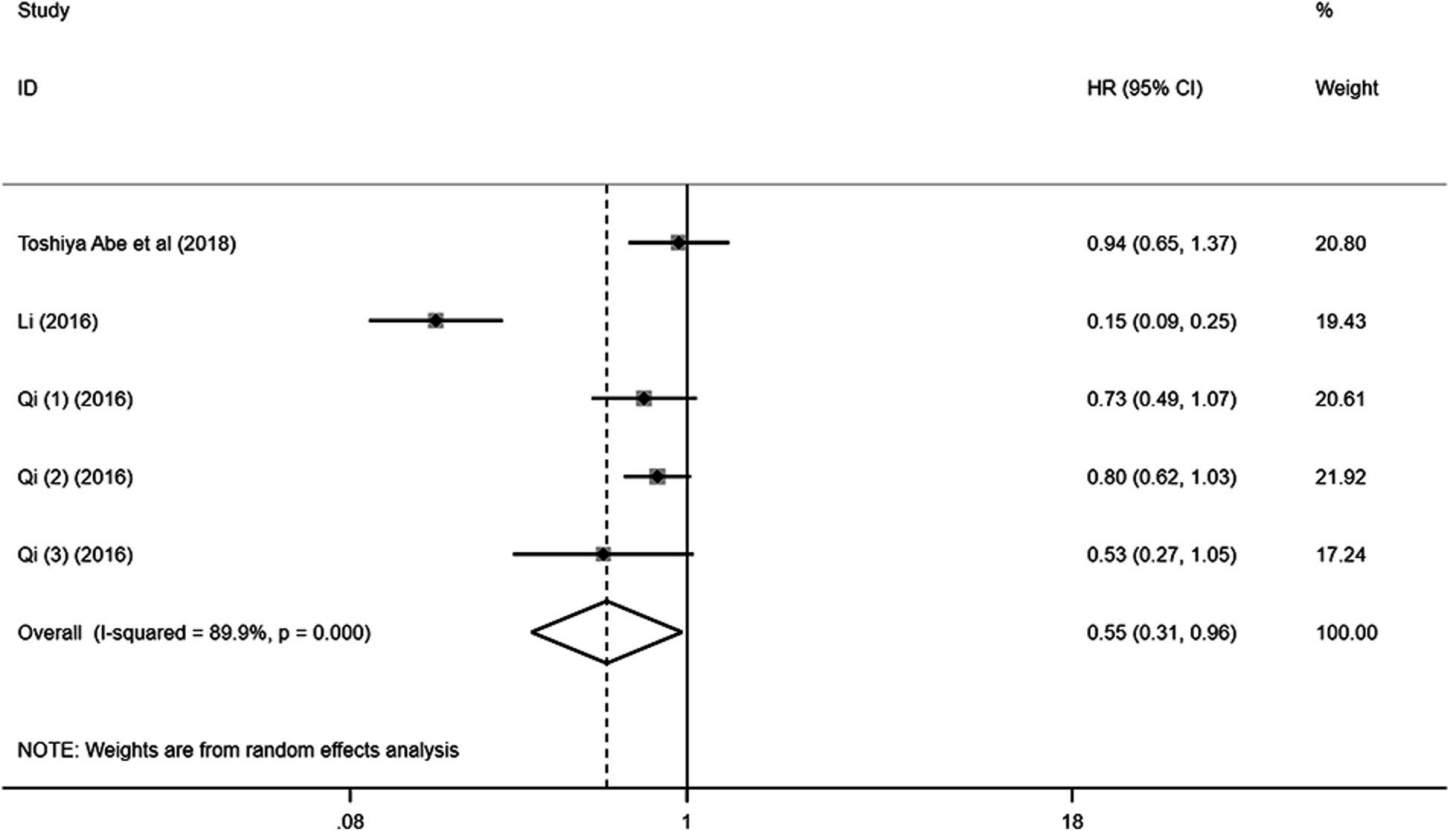
A meta-analysis of the association between LMR and DFS/RFS/TTP of pancreatic cancer. Results are presented as individual and pooled hazard ratios (HRs) and 95% confidence intervals (CIs)

Barely 5 cohorts reported HRs for DFS/RFS/TTP in PC patients. Data from the random-effects model showed significant heterogeneity (*I*-squared = 89.9%, Ph < 0.001). Analysis of the pooled results from the random-effects model indicated that elevated LMR was significantly associated with better DFS/RFS/TTP in patients with PC (HR = 0.55, 95% CI 0.31–0.96, *P* = 0.037) (Fig. 3).

### Sensitivity analysis and publication bias

**Fig. 4:**
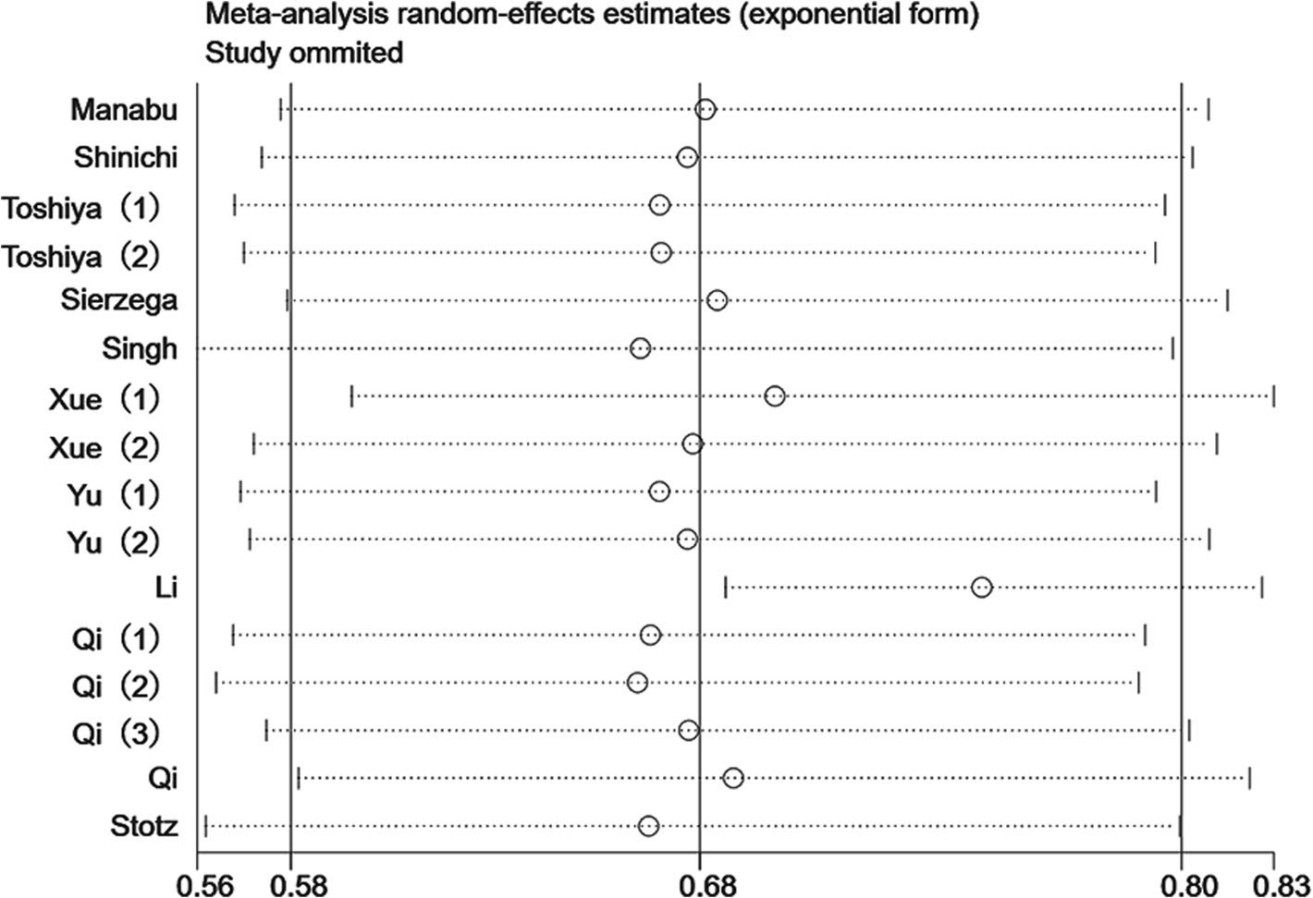
Chart showing sensitivity analysis

We further performed a sensitivity analysis to evaluate the stability of the calculated results. Results revealed that the pooled HR was not significantly affected by any independent study; this confirmed the stability and reliability of our data (Fig. 4).

In addition, publication bias was assessed by Begg’s funnel plot and Egger’s linear regression test. Our meta-analysis results demonstrated no significant publication bias (*P* > |*z*| = 0.260 for Begg’s test and *P* > |*t*| = 0.142 for Egger’s test).

## Discussion

Pancreatic cancer, a malignant tumor, is the 7th leading cause of cancer-related deaths globally. The diagnosis and treatment of pancreatic cancer are problematic, with a 5-year survival rate close to 5% [2]. Despite the success rate and the constant enhancement of the prognostic tools over the years, the prognosis of patients with pancreatic cancer has not improved correspondingly. Therefore, there has been an urgent need to develop sensitive and specific biomarkers to predict the prognosis of PC patients. Previous research demonstrated that several tumor-related indicators including inflammation-related markers and tumor properties histological features highly contributed to the progression of cancer, immunosuppression, metastasis, and establishment of pre-cancer niche [16, 41]. So far, pretreatment LMR, a systemic inflammatory biomarker, has been revealed to be associated with prognosis in PC patients [26–28, 39]. However, the exact relationship between pretreatment LMR and survival outcome of pancreatic cancer remains unclear.

Results from previous meta-analyses have reported that elevated LMR is associated with better OS and DFS/RFS/TTP in patients with PC [32, 33]. Nevertheless, these meta-analyses involving all studies published before 2017 utilized a relatively small sample size. Moreover, validation cohorts in some studies have not been captured, which may cause heterogeneity and publication bias [31, 38, 39]. Also, the pooled results showed significant heterogeneity with distinctive findings from subgroup analyses between studies. Therefore, reconsideration should have been at play when new pieces of literature were being published over time [29, 30, 34]. In our present meta-analysis, we pooled the outcomes of 11 studies (16 cohorts) with 3338 patients and assessed the prognostic value of LMR in pancreatic cancer. The final result demonstrated that elevated pretreatment LMR was significantly associated with better OS and DFS/RFS/TTP in PC patients. Furthermore, subgroup analyses revealed that high LMR was significantly associated with better OS in PC patients irrespective of ethnicity, study design, treatment, variable type, and a cut-off value of LMR. Additionally, our findings corroborated with previous studies by highlighting that elevated LMR may have a better prognostic role for OS in patients with advanced and mixed disease, particularly those with advanced disease [32, 33]. Studies by Hu et al. and Li et al. validated our findings whereby they asserted that the serum level of LMR was negatively associated with the tumor stage progression in PC patients [32, 33]. Also, through pooled results, we further found that high pretreatment LMR predicted a better OS in PC patients regardless of univariable or multivariable analyses. Finally, sensitivity analysis demonstrated that our results were relatively stable while the Begg’s funnel plot and Egger’s linear regression test reported no significant publication bias in this meta-analysis. Conclusively, LMR may be considered as an important biomarker for the prognosis of PC. However, as shown in our results, and unlike some studies, the cut-off value of LMR varied and ranged from 2.05 to 4.6. It is worth noting that most studies calculated their cut-off value using receiver operating characteristic curves (ROC), and a standard method of selecting the cut-off value is still unclear in the others. Elsewhere, a study conducted by Koh et al. reported that the cut-off value of LMR was linked to the age of patients [42]. Therefore, it is conceivable that the optimal cut-off value must be adjusted following the currently unknown clinical and pathological features and/or by each tumor entity on its own. Besides, the large number of Asian patients included in this meta-analysis compared to Caucasians. Despite this may expose the difference in genetic background among different races [43], as well as differences in the environment and the lifestyle between Asian and Caucasian. According to the 2018 global cancer statistics, incidence rates of PC are higher in developed countries compared to developing countries with the highest incidence rates reported in Europe, North America, and Australia/New Zealand. This suggests that the incidence rates of PC in Caucasians are higher compared to Asians [2]. Additionally, as our knowledge, research parameters have not shown any significant difference in the treatment strategy of PC and lifestyle aspects linked to either LMR or PC between Asians and Caucasians. Owing to the large number of Asian studies included in this meta-analysis, it is more suitable for the Asian populations and their background; thus, there is a need for further large-scale and meticulously designed studies to verify the prognostic value of pretreatment LMR in different races. What is more, the pooled results revealed significant heterogeneity; thus, they should be interpreted with a disclaimer. Therefore, further studies with a large sample size and more details of the subjects are essential to substantiate our findings.

The definite mechanisms of the link between pretreatment LMR and survival outcome in PC patients remain unclear. However, reports suggest that tumor-related inflammation contributes to multifactorial function in tumorigenesis and the development of PC [15, 44]. Despite the elusive details on the causal relationship between inflammation and cancer [45], previous studies showed that inflammation in the tumor microenvironment promotes tumor spread and lower the responses to anticancer drugs, eventually affecting proliferation and survival rate of cancer cells. Presently, researchers have been prompted to increasingly focus more attention on the crucial and multifarious role of inflammation in tumorigenesis and progression of malignant tumors [15, 17, 45]. Inflammation-related prognostic scores utilizing blood count parameters, for example, LMR, PLR, and NLR, have been progressively identified in diverse malignant tumors, including PC. Notably, lymphocytes play a critical part in cellular immunity, including CD4^+^ and CD8^+^ cells, especially tumor-infiltrating T lymphocytes, which suppress the proliferation and migration of tumors through apoptosis [46]. The number of peripheral blood lymphocytes corresponds to the state of immunity in the body. Studies have also reported lymphocytopenia as a novel indicator in the prognosis of patients with PC [47, 48].

In the tumor microenvironment, monocytes could potentially differentiate into tumor-associated macrophages (TAMs) promoting angiogenesis, extracellular matrix remodeling, tumor invasion, and metastasis by secreting epidermal growth factor (EGF), vascular endothelial growth factor (VEGF), interleukin-6, interleukin-10, and metalloproteinase [49]. Furthermore, TAMs upregulate the programmed cell death 1 (PD-1) expression forming a local immunosuppressive microenvironment, thus facilitating the immune escape of cancer cells [50]. Also, findings from previous research work argued that TAMs were associated with poor survival in many malignant tumors [51, 52]. Therefore, the potential of LMR as a prognostic biomarker in PC is demonstrated by high lymphocyte count and low monocyte count which may reflect systemic inflammation that inhibits tumor progression and migration.

From this study, we collected some common strengths of the enrolled studies. All enrolled cohorts reported the association between LMR and OS in patients with PC. And, some of the enrolled studies worked with a large sample size, long follow-up period, and multidisciplinary data. Besides, 4 studies set validation of the prognostic value of the LMR in an independent cohort [29, 31, 38, 39], which was key in generalizing the findings of such biomarkers according to the REMARK criteria [53, 54]. However, there were several limitations of our meta-analysis. Firstly, only 16 cohorts with 3338 subjects were enrolled in this meta-analysis; hence, our results might be affected by a relatively small sample size. Secondly, significant heterogeneity could not be excluded among the eligible studies; therefore, a random-effects and subgroup analyses were conducted to increase the homogeneity of the study. It is a common scenario to find heterogeneity between studies when a meta-analysis of observational studies is conducted. Also, the differences in the quality of studies, study design, the country, variable type, sample size, tumor stage, cut-off value, and the method of treatment in our meta-analysis might be the probable sources of heterogeneity. In subgroup analyses based on tumor stage, only studies with stages III–IV and I–IV revealed a significant association between pretreatment LMR and a better OS. This means that pretreatment LMR may be of more significant prognostic value in advanced pancreatic cancer. Moreover, sensitivity analysis showed that the results of our meta-analysis were stable. Again, most of the enrolled cohorts were retrospective, and data from prospective studies were insufficient. Large-scale multicenter prospective cohorts are required to clarify the prognostic value of pretreatment LMR in PC. This study was restricted to literature reported in English so that publication bias cannot be completely excluded. Finally, the cut-off value was different among the studies, which led to a significant change in HR. We speculate that it may be due to the inconsistent number of patients in groups hence changing the cut-off value, and resulting in different HR. In future studies, the optimal cut-off value of LMR should be validated for advanced research needs and clinical applications.

## Conclusion

In conclusion, our meta-analytic findings suggested that elevated pretreatment LMR is associated with better OS as well as low risk of recurrence in patients with PC. In addition, we found that pretreatment LMR may act as a novel biomarker for the prognosis of PC patients. Due to the limitations of this meta-analysis, further large-scale, multicentered, well-designed, prospective, randomized, and controlled analyses are essential to validate the optimal cut-off value and prognostic role of pretreatment LMR in patients with PC.

## Supplementary information

**Additional file 1: Figure S1.** A meta-analysis of the association between pretreatment LMR and overall survival (OS) of pancreatic cancer based on univariable analyses. Results are presented as individual and pooled hazard ratios (HRs), and 95% confidence intervals (CIs).

**Additional file 2: Figure S2.** A meta-analysis of the association between pretreatment LMR and overall survival (OS) of pancreatic cancer based on multivariable analyses. Results are presented as individual and pooled hazard ratios (HRs), and 95% confidence intervals (CIs).

## Data Availability

The datasets used and/or analyzed during the current study were availed by the corresponding author on reasonable request.

## Abbreviations

CI: Confidence interval
DFS: Disease-free survival
HR: Hazard ratio
PC: Pancreatic cancer
OS: Overall survival
PFS: Progression-free survival
TTP: Time to progression
LMR: Lymphocyte to monocyte ratio
