# Supplementary figures and images for: Prognostic value of lymphocyte to monocyte ratio in pancreatic cancer: a systematic review and meta-analysis including 3338 patients
Source: World J Surg Oncol. 2020 Jul 25;18:186. doi: 10.1186/s12957-020-01962-0 (PMC7382838; doi:10.1186/s12957-020-01962-0)

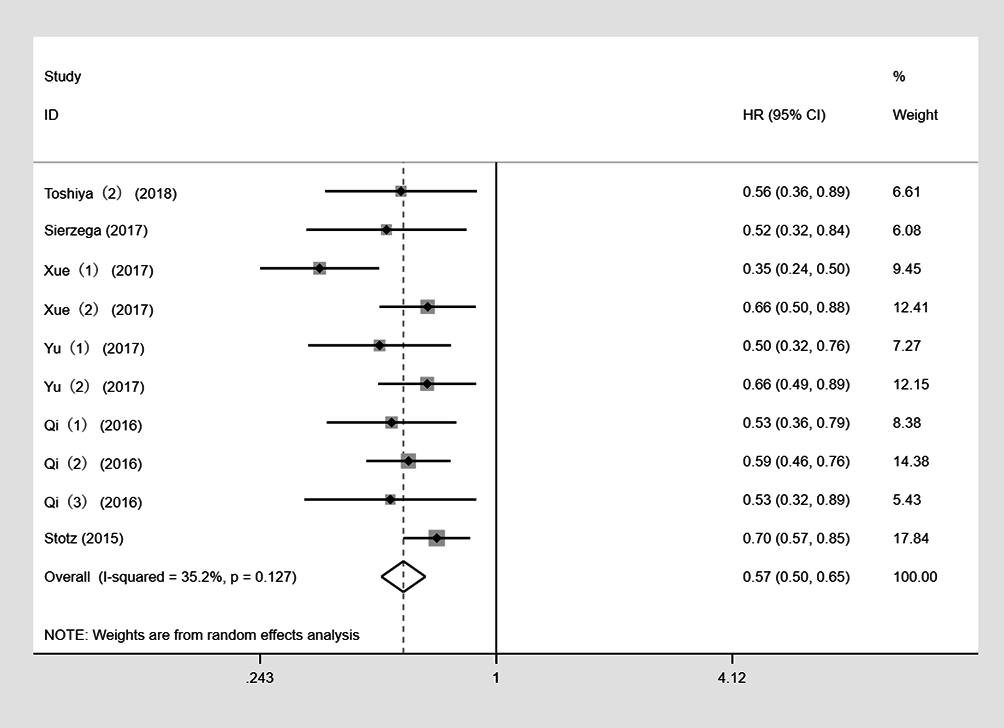

Supplement: Supplementary file 1 — Additional file 1: Figure S1. A meta-analysis of the association between pretreatment LMR and overall survival (OS) of pancreatic cancer based on univariable analyses. Results are presented as individual and pooled hazard ratios (HRs), and 95% confidence intervals (CIs). [file 12957_2020_1962_MOESM1_ESM.tif]

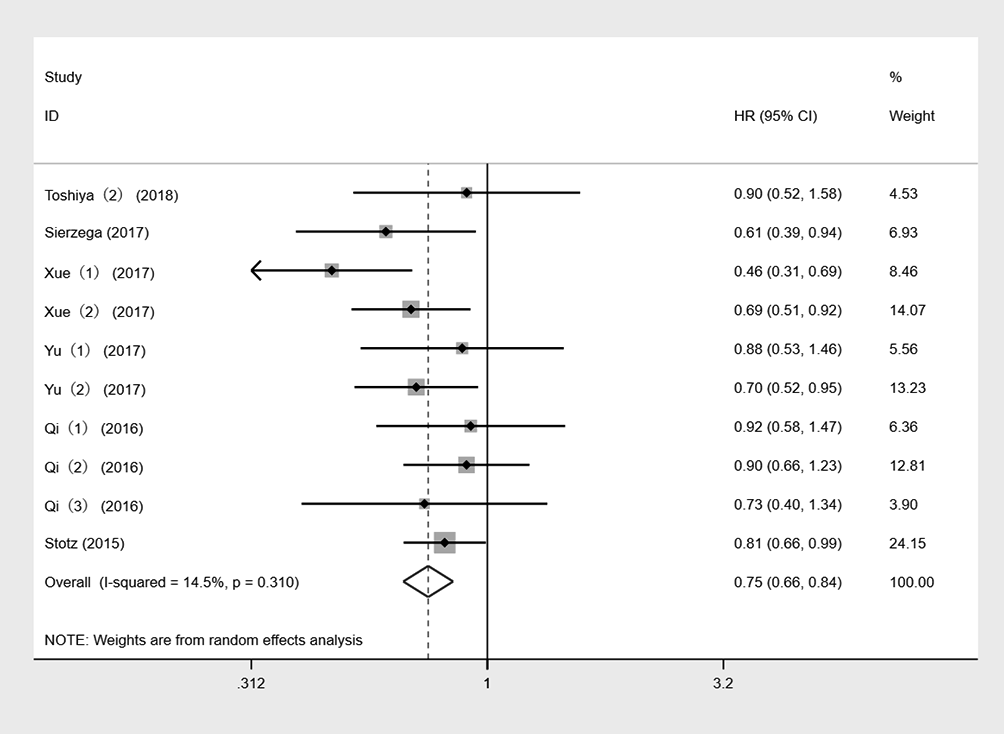

Supplement: Supplementary file 2 — Additional file 2: Figure S2. A meta-analysis of the association between pretreatment LMR and overall survival (OS) of pancreatic cancer based on multivariable analyses. Results are presented as individual and pooled hazard ratios (HRs), and 95% confidence intervals (CIs). [file 12957_2020_1962_MOESM2_ESM.tif]
